# Amphiphiles capsaicin and triton X-100 regulate the chemotherapy drug colchicine’s membrane adsorption and ion pore formation potency^☆^

**DOI:** 10.1016/j.sjbs.2021.02.054

**Published:** 2021-02-21

**Authors:** Md. Ashrafuzzaman

**Affiliations:** Biochemistry Department, Science College, King Saud University, Riyadh 11451, Saudi Arabia

**Keywords:** Chemotherapy drug, Amphiphiles, Membrane, Ion pore

## Abstract

Chemotherapy drugs (CDs), e.g. colchicine derivative thiocolchicoside (TCC) and taxol, have been found to physically bind with lipid bilayer membrane and induce ion pores. Amphiphiles capsaicin (Cpsn) and triton X-100 (TX100) are known to regulate lipid bilayer physical properties by altering bilayer elasticity and lipid monolayer curvature. Both CDs and amphiphiles are predicted to physically accommodate alongside lipids in membrane to exert their membrane effects. The effects of their binary accommodation in the lipid membrane are yet to be known. Firstly, we have performed experimental studies to inspect whether membrane adsorption of CDs (colchicine or TCC) gets regulated due to any membrane effects of Cpsn or TX100. We find that the aqueous phase presence of these amphiphiles, known to reduce the membrane stiffness, works towards enhancing the membrane adsorption of CDs. Our recently patented technology ‘direct detection method’ helps address the membrane adsorption mechanisms. Secondly, in electrophysiology records, we measured the amphiphile effects on the potency of ion channel induction due to CDs. We find that amphiphiles increase the CD induced channel induction potency. Specifically, the membrane conductance, apparently due to the ion channel induction by the TCC, increases substantially due to the Cpsn or TX100 induced alterations of the bilayer physical properties. Thus we may conclude that the binary presence of CDs and amphiphiles in lipid membrane may influence considerably in CD’s membrane adsorption, as well as the membrane effects, such as ion pore formation.

## 1 Introduction

Chemotherapy drugs (CDs) induce ion pores inside lipid bilayer membranes (Ashrafuzzaman et al., 2011; Ashrafuzzaman et al., 2012). Using electrophysiology (EP) records we observed thiocolchicoside (TCC) and taxol (TXL) to induce pores inside model membranes under an applied transmembrane potential. These pores resemble the structure of toroidal channels (Ludtke et al., 1996, Matsuzaki et al., 1996).

The popular CD candidates colchicine (Col) and taxol (TXL) derivatives are generally investigated to address their possible applicability as drugs. Colchicine has a long history of use in immune-system diseases (Seideman et al., 1987, Callen, 1985). It is found to bind with nuclear periphery and to disorder the nuclear membrane phospholipid bilayers (Agutter and Suckling, 1982). TXL, incorporated into liposomes, is found to penetrate into the acyl chain domain which alters physical properties e.g., phospholipid phase transitions, lipid order parameters, fluidity etc. of membranes (Balasubramanian and Straubinger, 1994). It is therefore not surprising to see CDs to alter bilayer conductance properties by especially inducing ion pores there (Ashrafuzzaman et al., 2011, Ashrafuzzaman et al., 2012).

*In silico* molecular dynamic (MD) simulations revealed considerable CD-lipid binding energetics, which perhaps is the reason behind recently observed membrane adsorption of CDs in *in vitro* assays (Ashrafuzzaman and Tseng, 2016, Ashrafuzzaman et al., 2020). These physical drug-lipid binding energetics and membrane adsorption mechanisms are predicted as the molecular mechanisms behind CD induced toroidal pore formation across lipid bilayers. A toroidal pore, induced by CD candidate molecule (e.g. TCC) (Ashrafuzzaman et al., 2011, Ashrafuzzaman et al., 2012), generally requires two lipid monolayers to meet each other at the opening of the pore. Versatile ion channels, such as β-helical, toroidal type, barrel-stave pores, etc. are induced in lipid membranes by various antimicrobial peptides (AMPs) and other drugs like our reported CDs (Boheim, 1974, Andersen, 1983, Andersen, 1984, Ludtke et al., 1996, Matsuzaki et al., 1996, He et al., 1996, Ashrafuzzaman et al., 2012).

**Fig. 1:**
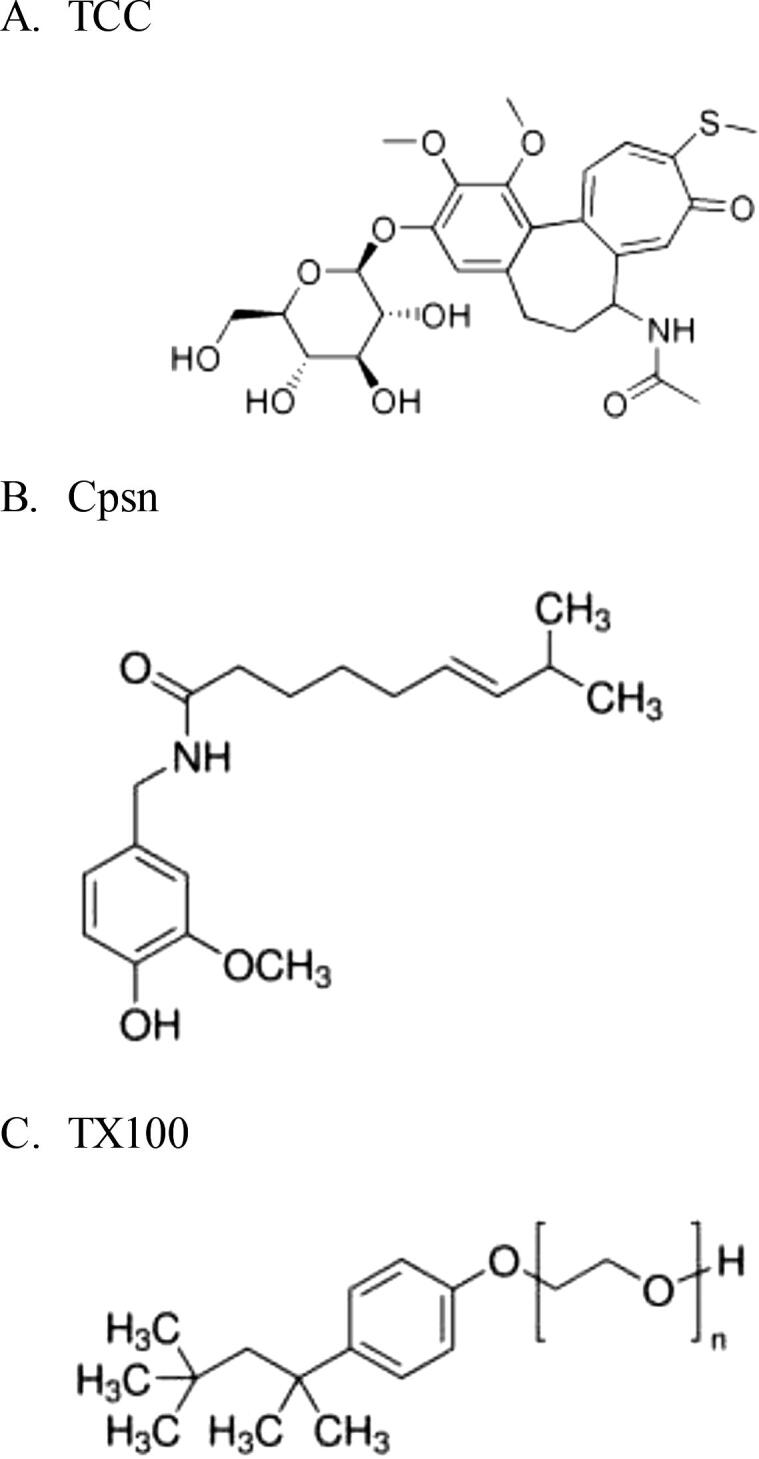
**.** A. Top panel presents the TCC structure (A). Cpsn (B) and TX100 (C) structures are copied from Sigma.

The CD pores are recently found to get regulated due to the effects of a cationic cyclic decapeptide gramicidin S (GS) at concentrations below its defect forming potency (Ashrafuzzaman et al., 2008a, Wu et al., 1999), see supp. Fig. 1 (Ashrafuzzaman, submitted). Two types of GS effects have been reported here: GS induced enhancement of membrane adsorption of CD molecules and membrane induction of CD pores. GS, negative lipid curvature profile promoting AMP (Staudegger et al., 2000), is found to stabilize toroidal CD pores (Ashrafuzzaman, submitted) and peptide channels, e.g. β-helical gramicidin A (gA) gA and barrel-stave alamethicin (Alm) channels (Ashrafuzzaman et al., 2008b) in lipid bilayer, as like as amphiphiles capsaicin (Cpsn) and triton X-100 (TX100) (see their structures in Fig. 1) which are found to stabilize gA channels (Lundbaek et al., 2005) and Alm channels (Ashrafuzzaman et al., 2007) (see supp. Fig. 2 and supp. Fig. 3). Cpsn is an active chili pepper component while TX100 is a non-ionic detergent. Cpsn and TX100, negative and positive lipid curvature profile promoting amphiphiles, respectively (Lundbaek et al., 2005) have been found to reduce the lipid bilayer stiffness by altering the lipid bilayer elasticity and the intrinsic curvature and thus upregulate the bilayer hosted channel functions (details will appear in another manuscript by Ashrafuzzaman and Andersen-to be submitted) (Ashrafuzzaman et al., 2007). All these three agents GS, Cpsn and TX100 are predicted to universally stabilize ion channels or pores by perhaps primarily reducing the bilayer stiffness. We wished here to address the role of these amphiphiles Cpsn and TX100 on (a CD molecule) TCC pore functions in model membrane systems. We have addressed here two specific physical aspects related to the quantification of the amphiphile induced possible alterations of (i) membrane adsorption of TCC molecules and (ii) the TCC induced membrane conductance. Former one is demonstrated using our recently patented technique ‘direct detection method (DDM)’ helping to measure the target structure (liposome) adsorbed mole fraction of drugs dissolved in liposome bathing aqueous phase (Ashrafuzzaman and Tseng, 2016, Ashrafuzzaman et al., 2020). Latter one is demonstrated using the popular EP record of membrane current technique (Ashrafuzzaman et al., 2006, Greisen et al., 2011). Both of these demonstrations help us address how drug effects on membrane may get regulated due to general membrane active agents (MAAs), no matter whether they form specific membrane conducting pores or/and alter membrane’s general physical or chemical properties. This study may open up new ways to regulate the CD effects on specific cell membrane physical properties and general cytotoxicity (unwanted damage to normal cells caused by anticancer drugs) which is one of the major issues to be tackled during chemotherapeutic applications of anticancer drugs (Muvaffak et al., 2004, Lin et al., 2016).

## 2 Materials and methods

### 2.1 Electrophysiology Experiments:

We have utilized a lipid cocktail of phosphoethanolamine: phosphatydyleserine: phosphatidylcholine (5:3:2, v/v/v)/n-decane and formed planar lipid bilayers over a 150 µm septum on the bilayer cuvette. The aqueous volume of the cuvette was maintained at 1 mL, which was filled with an aqueous buffer containing 0.5 M NaCl, 10 mM HEPES at pH 7.4 in both of the chambers. The *cis* side was connected with the recording electrode and the *trans* side with the reference electrode.

The stock of TCC (collected from ChemRoutes, Canada) was prepared in dimethylsulfoxide (DMSO) at 4 mg/mL or 7.1 mM concentration, and diluted in the buffer for further experimental use at concentration 1 mg/mL. Cpsn and TX100 were purchased from Sigma.

Following the bilayer formation we waited for a period of about 1 h to test and ensure confirmation of the bilayer stability by applying a high transmembrane potential (V), around V = 400 mV. After rigorous vortexing, an aliquot of ion pore forming agent TCC stock was added to the cis side of the chamber buffer, while stirring to avert any issues related to the stability of the drug. Usually after about 20 min of the addition of TCC, we started recording currents at various applied transmembrane voltage. Long time (~60 sec) traces were recorded. The membrane current demonstrates occasional nonzero values, representing the statistical appearance of conductance events, due to formation of TCC induced ion pores across the lipid bilayer (Ashrafuzzaman et al., 2011; Ashrafuzzaman et al., 2012). To investigate the effects of amphiphiles on TCC pore forming mechanism, we then added aliquot of Cpsn or TX100 stock in both chambers that is on both sides of the bilayer. The concentrations of Cpsn and TX100 used here are known, in earlier studies (Lundbaek et al., 2005, Ashrafuzzaman et al., 2007, Greisen et al., 2011), not to alter the bilayer’s gross electrical insulation properties. Following the addition of amphiphiles, we waited for about 20 min and conducted recording of current traces across the lipid bilayer under the influence of considerable transmembrane potentials. At the least we performed three experimental repeats, so that we could demonstrate statistical nature of the effects.

For performing EP records, we recently established a lipid bilayer workstation at our biophysics laboratory which was provided and installed by Warner Instruments LLC, 1125 Dixwell Av., Hamden, CT 06514. DIGIDATA 1440 (low-noise data acquisition system) and pCLAMP 10 CNS software for Windows were provided by Molecular Devices (UK) Ltd. We just mimicked the EP records in refs. (Ashrafuzzaman et al., 2011; 2012). Membrane current (in pico ampere (pA) scale) was recorded at the filter frequency 20 kHz (Ashrafuzzaman et al., 2006; Ashrafuzzaman et al., 2012). The program Origin 9 (OriginLab Corp., Roundhouse Plaza, Suite 303, Northampton, MA 01060, USA, www.originlab.com) was used to plot the current versus recorded time.

### 2.2 DDM-based experiments

DDM was recently developed and applied to detect various lipid-bound MAAs in mole (M) fraction. We already addressed DDM for detecting CDs *in vitro* DOPC and aptamers *in vitro* DPPC and DPPS liposome systems using standard absorbance spectroscopy (for details see ref. (Ashrafuzzaman and Tseng, 2016, Ashrafuzzaman et al., 2020). Existing techniques, e.g. EP record of current, fluorescence measurements, etc. address the general lipid binding effects, whereas the DDM helps detect the molecules directly at their target site(s). The main rinciple behind using DDM is that we separate the lipid bound and unbound MAAs in solution and then detect the MAAs in these separated solutions. Let us denote B and UB for the solutions separated as mole fraction of MAAs bound to lipids and unbound ones, respectively. We then use a NanoDrop (purchased from ThermoFisher Scientific) or a Nanophotometer (purchased from Implen GmBH) to get the absorbance spectra that is specific for certain MAA. The wavelength (λ_MAA_) of the spectrum is actually MAA specific. λ_DNA_ = 260 nm is for DNA aptamers, λ_colchicine_ = 243 nm is for CD colchicine, etc. (please see the Sigma-Aldrich manual). We then perform the spectroscopy on both samples and quantify the concetrations of MAAs dissolved in both samples B and UB (Ashrafuzzaman et al., 2016; 2020). Using these detected concentrations, we calculate the molarities of both of the lipid-bound and lipid-unbound MAAs in incubation tube. These concentrations are then normalized with correct volume of the aqueous buffer in which the liposomes were formed, then the liposomes were incubated with drugs before splitting the whole solution into B and UB. These lipid bound drugs are nothing but considered as the DDM detected liposome bound drugs that are plotted later in this article.

## 3 Results

Two sets of experimental studies were performed. In first set, we did EP record of CD induced membrane currents without and with the effects of either amphiphile Cpsn or TX100 on bilayer membrane. In second set, we applied DDM to address the quantitative membrane adsorption of CD molecules without and with the effects of Cpsn or TX100. Both have been presented here in details.

**Fig. 2:**
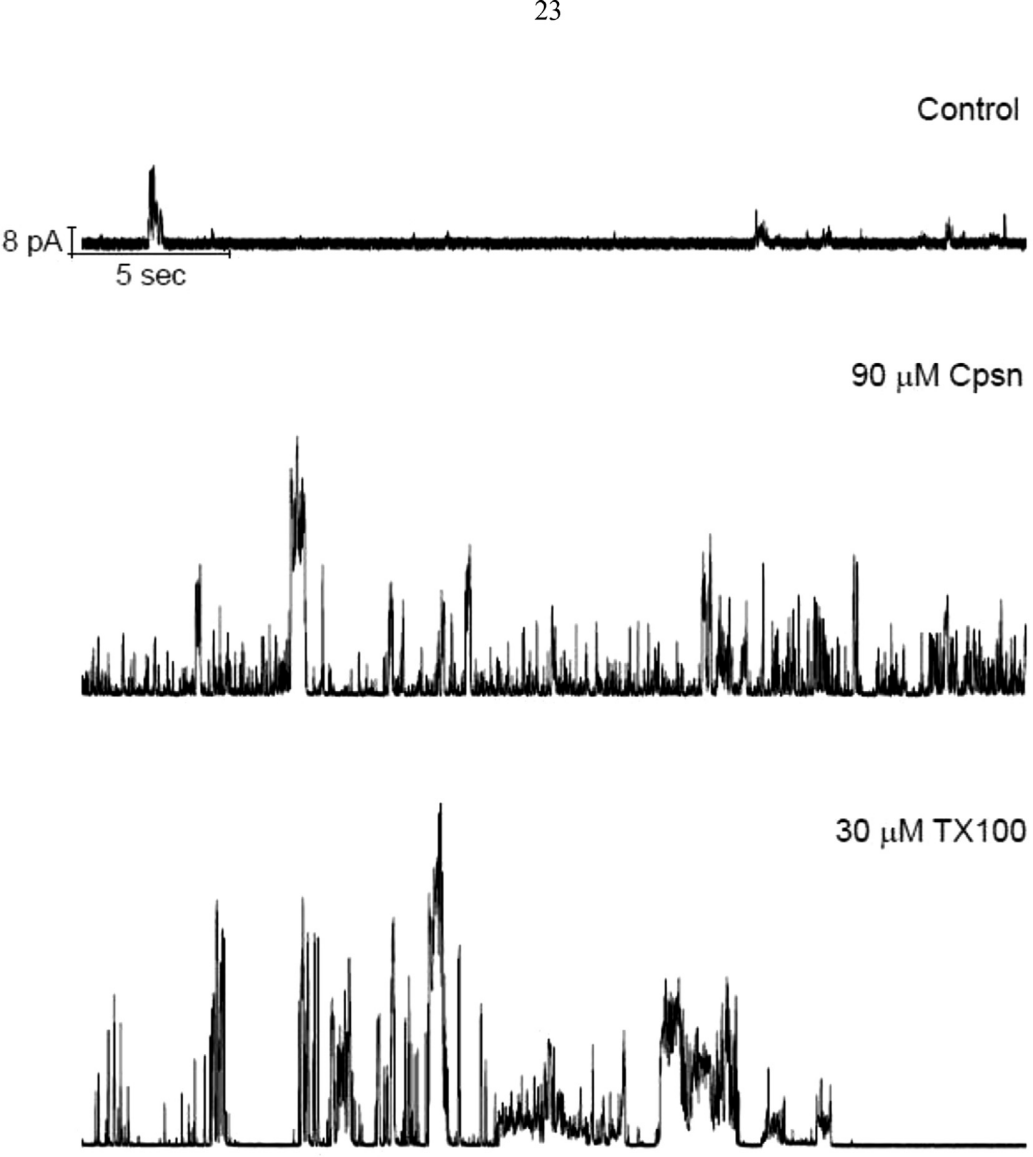
Cpsn or TX100 increases the CD’s ion pore induction potency. TCC (90 μM) alone permeabilizes lipid bilayer membranes by inducing nonzero current events (top panel). Middle and bottom panels represent the TCC induced current events being influenced by the effects of 90 μM Cpsn and 30 μM TX100, respectively, added into the aqueous phases in both chambers. Both traces were filtered at 20 kHz. The buffer in the cuvette was 500 mM NaCl containing 90 μM TCC at the *cis* side of the membrane and 0 μM TCC at the *trans* side of the membrane; 100 mV, pH 7.4.

**Fig. 3:**
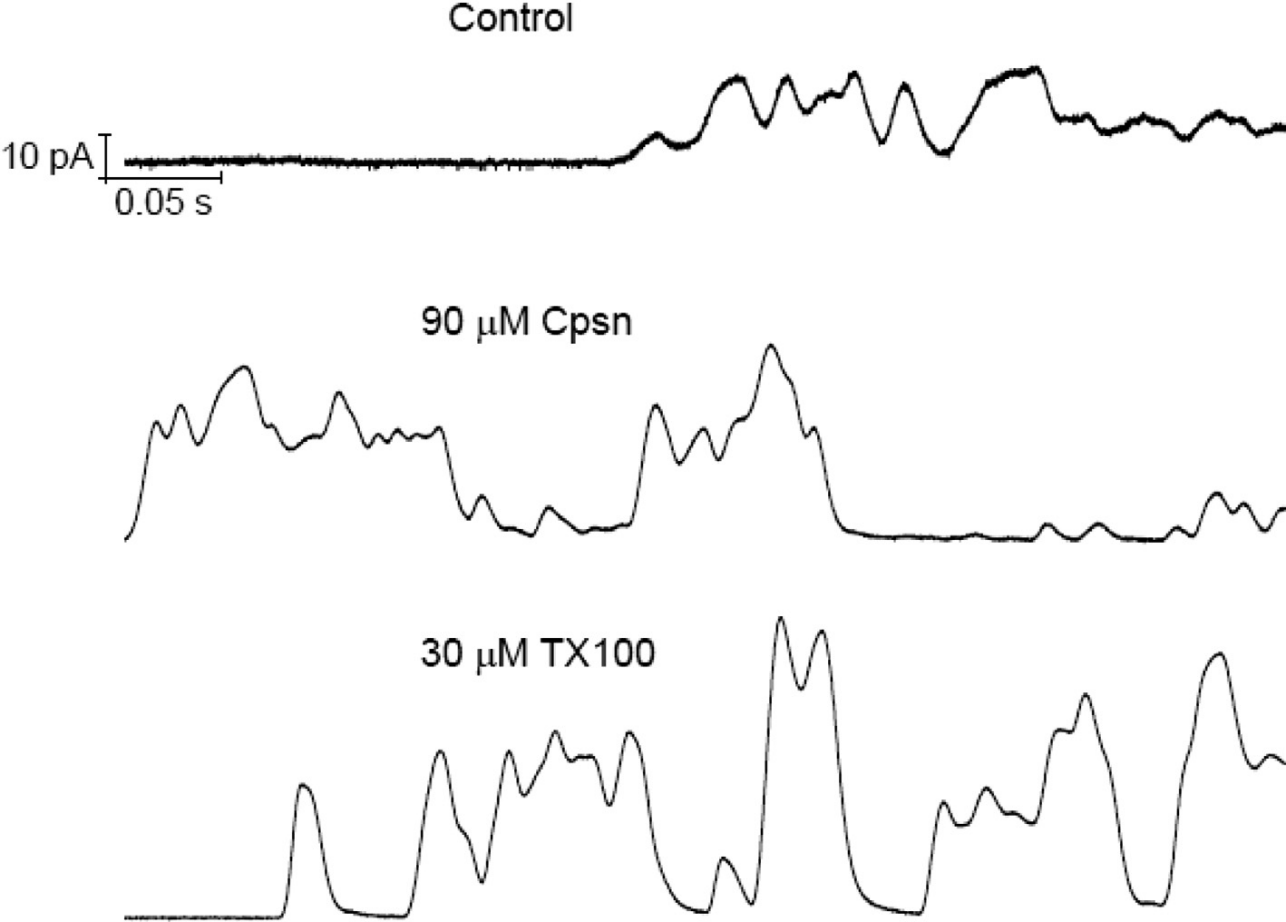
Short time (0.5 s) current traces. It is clear that the single channel current events resembles nothing but the triangular conductance events previously observed, see Fig. 4.

**Fig. 4:**
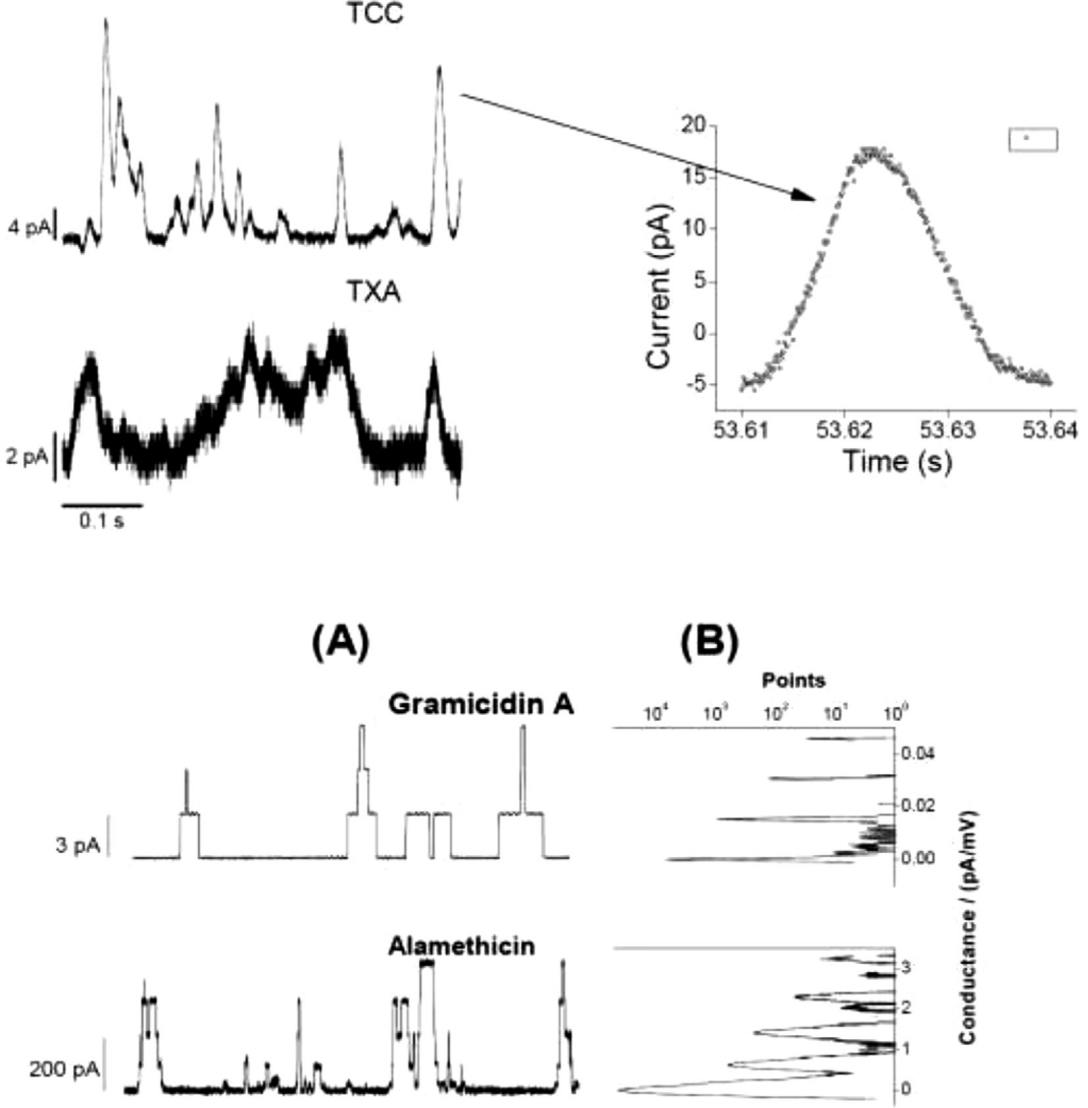
**.** Comparison of two CD TCC and taxol (TXA) pore current traces with peptide (AgA(15) and Alm) channel currents. The upper panel shows triangular-shape conductance events induced by TCC and TXL, both at 90 μM. pH = 5.7, *V* = 100 mV; both traces filtered at 20 kHz. In a high resolution plot (see at the right of the arrow) of a single event only with showing individual points (in Origin 8.5 plot) we observe all points (open circle) with increasing and decreasing, respectively, corresponding values of conductance at both left and right lateral sides of the CD induced triangular conductance events. The lower panel **(A)** illustrates rectangular-shape conductance events in gA and Alm channels (Ashrafuzzaman et al., 2008). gA channel activity was recorded at *V* = 200 mV and Alm at *V* = 150 mV. Here *I*_gA_ = 29 ± 2, 113 ± 5, 243 ± 9 and 386 ± 10 pA respectively are the discrete current levels 0, 1, 2, 3, …, etc in Alm channel. Traces representing gA and Alm channel activities in phospholipid bilayers were recorded at filter frequencies 2 kHz and 20 kHz, respectively. In **(B)** the point count plots of the current traces through AgA(15) and Alm channels. The peaks appear at discrete values of conductance for these two channels whereas for the CD pore conducting current traces there is only one delta (type) peak at 0 pA/mV conductance and the points at nonzero values of conductance are distributed all over without showing any specific or discrete conductance values due to the triangular nature of the CD pores (see top-right panel) (Ashrafuzzaman et al., 2011; 2012). The area under the peak at 0 pA/mV conductance of the membrane or 0 pA membrane current is nothing but the total point count proportional to the duration of the time when the membrane hosts no ion pores/channels. The sum of the areas under peaks at various nonzero conductance values (or for CD pores, sum of all points over all nonzero conductance values) is total point count proportional to the duration of time when the membrane conducts currents due to opening of the channels. For the purpose of comparison with previous data (Fig. 3), this Figure is taken from ref. (Ashrafuzzaman et al., 2012).

**Electrophysiology experiments demonstrating Cpsn or TX100 effects on TCC induced lipid bilayer currents.** Our earlier publications already demonstrated the EP records of lipid bilayer currents induced by two CD candidate molecules TCC and taxol (Ashrafuzzaman et al., 2011; Ashrafuzzaman et al., 2012). In this new set of experimental demonstration we find the effects of amphiphiles on CD induced pore currents across model membrane. Fig. 2 presents the Cpsn or TX100 effects on the CD pore currents. The concentrations of amphiphiles we have used here are previously found in various studies not to do any alterations in the gross lipid bilayer electrical insulation properties (Lundbaek et al., 2005, Ashrafuzzaman et al., 2007). Therefore, the observed changes in membrane current (through the appearance of increased number of conductance events) (reflected in Fig. 2, middle and bottom panels) over that of TCC induced currents (Fig. 2, top panel) are certainly not due to any effects of Cpsn or TX100 GS on bilayer electrical insulation. Moreover, in short-time trace analysis the recorded traces shown in current events (Fig. 3) resemble the patterns found in our earlier publications (Ashrafuzzaman et al., 2011; 2012). Fig. 3 presents the short-time current traces that show triangular conductance events in all of the cases without and with the effects of Cpsn or TX100. Thus it is suggestive that the observed conductance events are all due to TCC induced pores only. After careful analysis of CD pore currents in our previous publications we concluded the appearance of a new class ‘triangular conductance events’ that are toroidal type (Ashrafuzzaman et al., 2011; 2012). The single events of this new class of ion pore are compared with conductance events of two other highly studied ion channels, gramicidin A (gA) and alamtheicin (Alm) channels (Fig. 4).

**Fig. 5:**
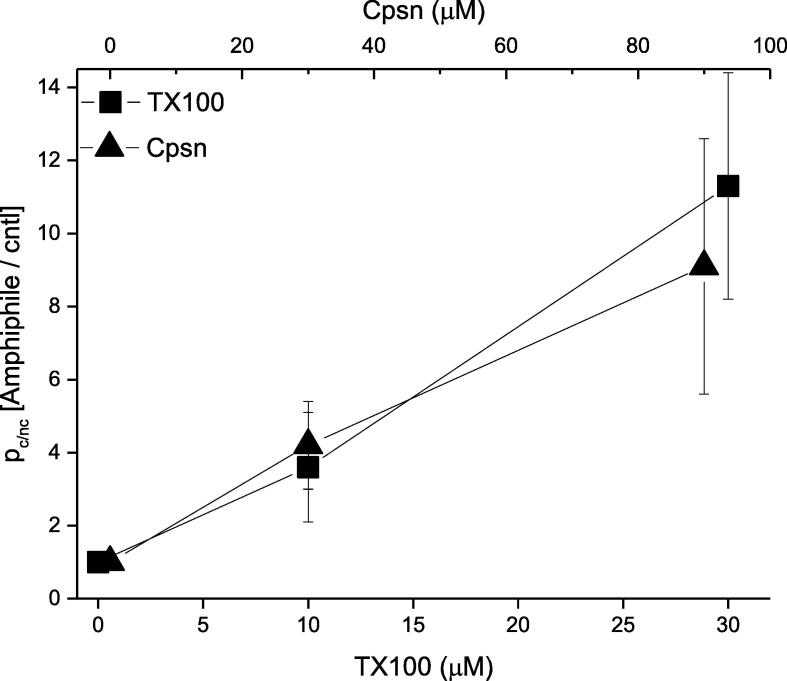
Effects of amphiphile Cpsn or TX100 on CD-induced pore opening probability: the membrane conductance probability relative to the membrane nonconductance probability *p*_c/nc_. Along y-axis is plotted the membrane conductance probability relative to the membrane nonconductance probability, being normalized by the value without the effects of amphiphiles (control condition). POPE:PS:PC = 5:3:2, 500 mM NaCl + 90 μM TCC (cis side of the membrane), 500 mM NaCl + 0 μM TCC (trans side of the membrane), 100 mV, pH 7.4. Cpsn or TX100 was added in the buffer on both sides of the bilayer.

We then performed the quantitative analysis of the conductance events. We observe increased appearance of conductance events with the increase of the buffer incubating concentration of Cpsn or TX100. The results are presented in Fig. 5 showing that the membrane conductance (due to the opening of CD pores) probability over the membrane nonconductance probability increases with the increase of Cpsn or TX100 concentrations, coexisted alongside the pore forming TCC in buffer. TX100 appears almost 3-fold more potent than Cpsn, like we found them exerting on other channels, namely gA and Alm channels in earlier studies (Ashrafuzzaman et al., 2007). Along y-axis (Fig. 5) is plotted the membrane conductance probability relative to the membrane nonconductance probability,

*p*_c/nc_ = Σ_i_t_c,i_/t_nc_ = Σ_i_t_c,i_/(Σ_i_t_c,i_ + t_nc_)/(t_nc_/ (Σ_i_t_c,i_ + t_nc_)) = membrane conductance probability/membrane nonconductance probability.

Here t_c,i_ is the time (in second) during when membrane conducts currents due to opening of i-th CD pore, i = 1, 2, …, n. Here we assume that the membrane holds n number of membrane conductance events as a result of the induction of n number of CD pores over the specified duration of (EP recorded) time, t_rec_ (in sec). The amount of time the membrane avoids conducting currents over the whole recorded time t_rec_ is t_nc_ = t_rec_-Σ_i_t_c,i_. The calculation of t_c,i_ (proportional to the total points under peak) follows from our earlier developed point count versus conductance plot method explained in ref. (Ashrafuzzaman et al., 2011; 2012) and shown in Fig. 4 (see rightmost panels for gA and Alm channel data).

**Fig. 6:**
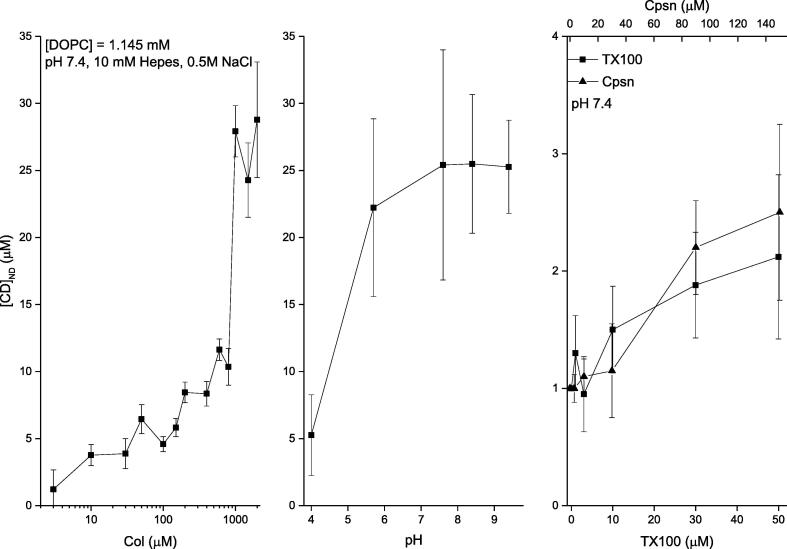
Three sets of data have been presented quantifying the DDM detected liposome bound colchicine for various aqueous environments bathing the liposome. Left panel: liposome bound colchicine as a function of various colchicine concentrations in aqueous phase. Middle panel: liposome bound colchicine as a function of various pHs in aqueous phase. Right panel: liposome bound colchicine (normalized by the value of liposome bound colchicine while buffer was incubated with 100 µM) as a function of various concentrations of two amphiphiles, Cpsn and TX100, in aqueous phase. pH and amphiphile effects have been investigated on liposome binding of colchicine while colchicine concentration in buffer [Col] = 100 µM. Different pH values have been obtained using the following conditions: pH 4, 5.7: 0.5 M Nacl + 20 mM acetate; pH 7.6, 8.4: 0.5 M Nacl + 20 mM Tris, pH 9.4: 0.5 M Nacl + 20 mM Glycine. Concentration of lipids in the cuvette/buffer has been maintained for all investigations at [DOPC] = 1.145 mM.

**Fig. 7:**
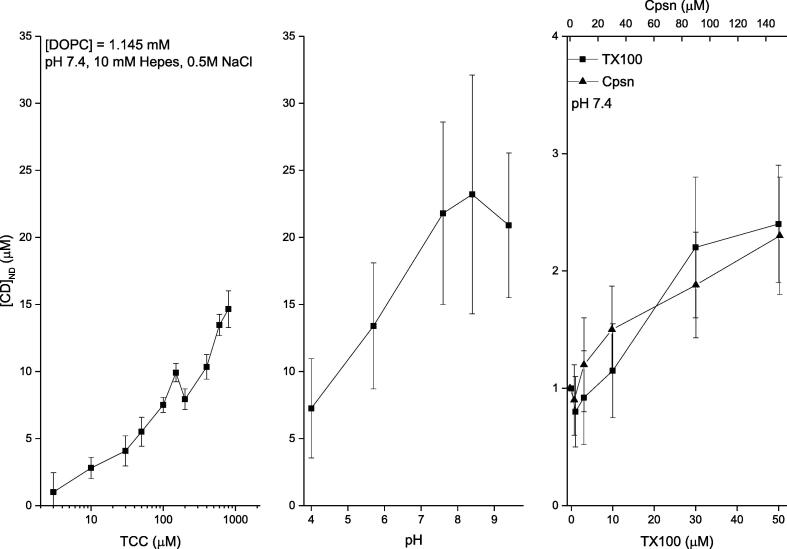
**.** Three sets of data have been presented quantifying the DDM detected liposome bound TCC for various aqueous environments bathing the liposome. Altough we could reach at equilibrium binding condition for Col (Fig. 6), we couldn’t reach at the equilibrium condition for TCC as I had only a modest amount of this derivative as leftover from our earlier studies (Ashrafuzzaman et al., 2011; 2012) what I utilized here. TCC is a very expensive derivative. Left panel: liposome bound TCC as a function of various TCC concentrations in aqueous phase. Middle panel: liposome bound TCC as a function of various pHs in aqueous phase. Right panel: liposome bound TCC (normalized by the value of liposome bound TCC while buffer was incubated with 100 µM) as a function of various concentrations of two amphiphiles, Cpsn and TX100, in aqueous phase. pH and amphiphile effects have been investigated on liposome binding of TCC while TCC concentration in buffer, [TCC] = 100 µM. Different pH values have been obtained using the following conditions: pH 4, 5.7: 0.5 M Nacl + 20 mM acetate; pH 7.6, 8.4: 0.5 M Nacl + 20 mM Tris, pH 9.4: 0.5 M Nacl + 20 mM Glycine. Concentration of lipids in the cuvette/buffer has been maintained for all investigations at [DOPC] = 1.145 mM.

**Fig. 8:**
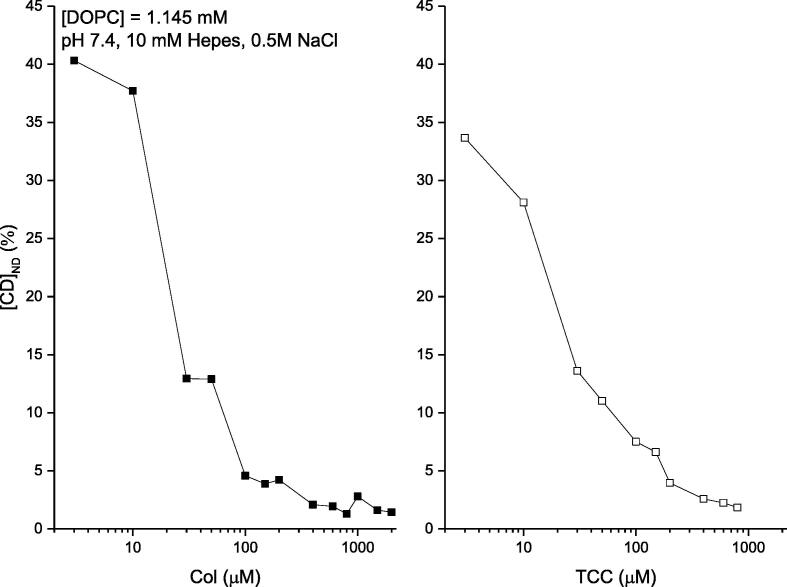
**.** Percentage (%) of liposome adsorbed CDs of their respective concentrations in buffer. These plots are deducted using the mean values presented in Figs. 6 and 7.

Cpsn or TX100 effects on the liposome binding of CDs dissolved in aqueous phase-DDM to detect agents directly at the liposome. The membrane adsorption of CDs is quantified *in vitro* liposome systems using DDM (Ashrafuzzaman and Tseng, 2016, Ashrafuzzaman et al., 2020). The phenomenological adsorption gets substantially regulated due to the environmental conditions of the aqueous buffer phase bathing the model liposomes. The experimental data are presented in Fig. 6, Fig. 7 (left panel) showing liposome adsorption potency of both Col and TCC, respectively, against their various aqueous concentrations bathing the liposomes. DDM method allowed us to NanoDrop (ND) detect the actual CD concentrations inside liposomes (plotted along y-axis) (Ashrafuzzman and Tseng, 2016; Ashrafuzzaman et al., 2020). Comparative analysis between data, plotted in Fig. 6, Fig. 7, suggests another important drug specificity aspect. As CD concentration in buffer increases, TCC starts showing modestly higher liposome adsorption potency reaching up to ~30% higher TCC binding over Col binding to liposomes. As we deduct the percentage of CDs adsorbed in liposomes relative to their respective buffer concentrations (Fig. 8), we observe that the binding potency decreases from around 40% at low CD concentration in buffer to as low as 1% at high CD concentration in buffer ~1 μM, and saturates beyond (tested for Col only). As the lipid concentration in the cuvette/buffer has been maintained for all investigations at [DOPC] = 1.145 mM, we could deduct the drug/lipid molar ratios in the liposome, which fall up to less than the value ~ 0.01 (for Col) and ~ 0.013 (for TCC). The value of the ratio suggests how much actual drug molecules from the aqueous phase physically get migrated into the vicinity of lipids, a process which certainly has important role for drugs in causing any specific or nonspecific effects on membrane regarding especially disrupting the membrane electrical insulation properties.

After addressing these control experiments, we planned on investigating the effects of Cpsn or TX100 in aqueous phase on liposome adsorption of CDs. Fig. 6, Fig. 7 (right panels) show the effects of amphiphiles on liposome binding potency of Col and TCC, respectively, at their 100 μM concentrations. Results suggest that both Cpsn and TX100 are potent regulators of liposome’s CD adsorption potency. The relative CD binding potency increases linearly with increasing Cpsn or TX100 concentration. We also observe that Cpsn effect is around 40% of the effects of TX100 on the CD molecules’ membrane adsorption potency, almost identical relative potency between these two amphiphies observed on their ion pore effects (Greisen et al., 2011, Ashrafuzzaman et al., 2007).

**pH effects on liposome binding of CDs.** The buffer pH effects on the liposome adsorption potency of both CDs, Col and TCC have been demonstrated. Fig. 6, Fig. 7 (middle panels) demonstrate the results. The liposome adsorption of both Col and TCC is found to increase with increasing pH at low pH value range (acidic condition). But as the neutral condition is met, the alteration in pH condition loses any considerable effects over a range of values of pH, heading towards the alkaline conditions. However, we need to consider that in the *in vitro* assays we had to use various chemical compositions for achieving different pH conditions in the experimental buffer.

## 4 Discussion

CD candidate molecules TCC and TXL were earlier reported by us to induce toroidal type ion pores inside lipid bilayers under applied transmembrane potentials. Amphiphiles Cpsn and TX100 are well known not to disrupt lipid bilayer’s electrical barrier properties but to alter other physical properties, such as the bilayer stiffness including the bilayer elasticity and curvature profiles. Like in other investigations (Ashrafuzzaman et al., 2007, Greisen et al., 2011), we also found here Cpsn to be almost 3-fold less potent than TX100 when it comes to act upon lipid bilayer membrane. This amphiphile induced alteration of bilayer physical properties appeared through inspecting the changes observed in functions of membrane hosted integral ion channels, such as gA and Alm channels. To address the possible universality of amphiphile effects on lipid bilayer, in current study we have inspected the effects of Cpsn and TX100 on membrane hosted toroidal type CD pores. Our results suggest overall similar responses CD pores draw, as like as gA and Alm channels, due to amphiphile effects on membrane (Ashrafuzzaman et al., 2007). On molecular level investigations, Cpsn and TX100 have already been reported to promote negative and positive curvatures in lipid structures in membranes, respectively (Lundbaek et al., 2005). Despite exerting opposite effects on the lipid curvature profiles, both were found to stabilize both gA and Alm channels inside lipid bilayers. Thus both of these amphiphiles are considered to reduce the general bilayer stiffness. Similarly, Cpsn and TX100 are both found in this study to increase the CD pore forming potency. Therefore, we wish to conclude that Cpsn and TX100 independently alter bilayer stiffness and lipid curvature profiles, thus promote ion channel function inside membranes. This statements are found valid for three different kind of channels, namely toroidal type (e.g. CD pore), β-helix (gA channels), and barrel-stave (Alm pores) structure forming channels. The current study further validates the universality of the effects of MAAs on bilayer properties that regulate membrane based versatile ion channels. MAAs, such as amphipathic molecules, amphiphiles, AMPs, amphiphiles, Cpsn, TX100, etc. were earlier reported to alter bilayer physical properties (reduces the bilayer stiffness) that regulates the integral ion channel functions (Lundbaek et al., 2005, Ashrafuzzaman et al., 2006; 2007; Greisen et al., 2011). Therefore, it is predictable that Cpsn and TX100 do something similar and thus regulate the CD pore functions.

To go dip inside, we performed another experimental study to address the amphiphile effects on actual liposome adsorption of ion pore forming MAAs. EP records on membrane currents across bilayer membrane doped with CD molecules suggest clearly that the membrane is naturally forced to accommodate agents that form ion pores inside, a key mechanism that is required to lead to compromising membrane’s electrical insulation properties. This mechanism requires considerable number of pore forming CD molecules to get adsorbed inside the membrane (Ashrafuzzaman et al., 2011; 2012). The membrane adsorption of CD molecules has quantitatively been addressed here using our patented DDM (Ashrafuzzaman and Tseng, 2016, Ashrafuzzaman et al., 2020). There is a considerable aqueous phase dissolved concentration dependence on membrane adsorption of the pore forming CD molecules observed. The DDM detection process of CDs directly at the membrane has been found to get considerably influenced due to the membrane effects of amphiphiles, at concentrations they are found to alter membrane physical properties (Lundbaek et al., 2005, Ashrafuzzaman et al., 2007, Greisen et al., 2011). The increase in membrane adsorption of CDs due to Cpsn or TX100 indirectly suggests that these amphiphiles perhaps exert considerable effects on the membrane accumulation potency of MAAs. Thus amphiphiles perhaps indirectly exert effects on pore formation of CDs inside lipid bilayers.

### 4.1 Modest effects of pH on both membrane adsorption of CDs and induction of CD pores

We reported in our earlier article that no considerable change in CD pore activity A was observed over a range of physiologically relevant pH values between 5.7 and 8.5, see supp. Fig. 4 (Ashrafuzzaman et al., 2011; 2012). Here A = Σ_i_t_c,i_/t_rec_.

Regarding comparable understanding of the buffer pH effects on CD adsorption and CD induced pore formation in lipid membranes, we find that both experimental observations help us draw almost identical conclusions. The liposome adsorption of both CDs Col and TCC is found to increase with increasing values of pH at low pH value range (acidic condition). But as the neutral condition is met, the alteration in pH condition loses any considerable effects over a range of values of pH, heading towards the alkaline conditions. But as mentioned earlier in methods section, these pH conditions in the aqueous phase have been maintained using varied chemical compositions which may also exert additional chemical-specific effects that we cannot extract here. Therefore, we may not be able to make any bold statement using these pH effects data. Probably, further comparable studies on drugs’ cytotoxicity assays using cancer and normal biological cells may help draw better understanding (not covered in this article, but we are investigating this issue using various healthy and cancer cell lines). Cancer cells physiologically exist in acidic conditions compared to the cells in normal physiological conditions (Persi et al., 2018). The acidification of extracellular region (low pH: 6.7–7.1) and alkalization of the intracellular cytoplasm (high pH ~ 7.2) are important hallmarks of cancer leading to a reverse gradient in pH across cancer cell membrane versus the trend found due to the corresponding values in normal cells, ~ 7.4 (extracellular region), and ~ 7.2 (intracellular cytoplasm region), respectively (Webb et al., 2011). Cancer cell targeted CDs may thus appear as very toxic for normal cells due that a bit higher CD adsorption potency is observed at neutral condition relative to the acidic condition. Therefore, these CD candidates may appear with substantial off-target toxicity potency, which is one of the major concerns many drug candidates need to tackle before appearing for clinical trials.

### 4.2 Amphiphile effects on CD pore formation and liposome adsorption are mutually correlated

**Fig. 9:**
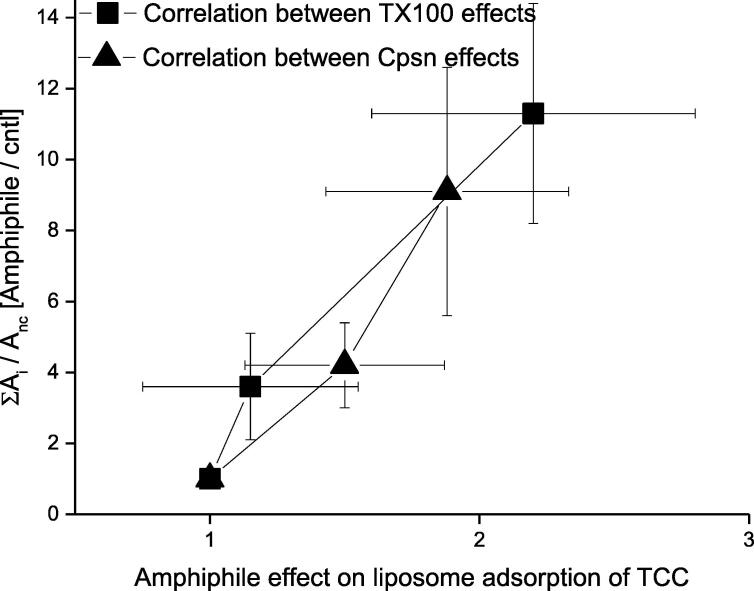
**.** Correlation between the effects of capsaicin ([Cpsn] = 0, 30, 90 μM) and TX100 ([TX100] = 0, 10, 30 μM) on TCC induced ion pore formation probability (y-axis) versus liposome adsorption potency of TCC (x-axis).

Fig. 9 summarizes important universal aspects of Cpsn and TX100 effects. Both TCC-induced pore formation and its liposome adsorption draw effects from the Cpsn or TX100 presence in the aqueous phase, which are mutually correlated. Although as Cpsn or TX100 concentration increases, the pore formation potency tends to draw relatively higher effects than the liposome adsorption. We, of course, can’t truly make any remarkable conclusion on this matter as we are limited within low amphiphile concentration range to avoid membrane from losing its integrity.

## 5 Conclusions

We suggest a few important findings. Firstly, the bilayer stiffness and lipid intrinsic curvature profiles altering apmphihphiles are found to regulate membrane hosted ion channels of versatile structures. Negative and positive lipid intrinsic curvature promoting amphiphiles Cpsn and TX100, respectively, stabilize CD pores identically as they stabilize gA and Alm channels in lipid bilayer membranes. Thus it is perhaps the bilayer stiffness that gets altered by both of these amphiphiles, and the membrane becomes less stiff, so all of these membrane hosted channels get stabilized. The effects of the reduction in bilayer stiffness perhaps dominates over the effects of (opposite type of) alterations in lipid curvature profiles, so we observe universal effects on functions of versatile channels hosted inside the membrane. Secondly, the quantitatively measured strong correlation between either amphiphile’s effects on membrane adsorption and ion pore forming potency of the CD molecules hints that the coexistence of MAAs with amphiphiles in the membrane vicinity (or inside the membrane) may influence each other’s (especially considered here the effects of amphiphiles on CD’s functions) effects on the membrane, depending on their relative concentrations, in various ways, such as influencing in membrane adsorption and accumulation inside, formation of stable structures, etc. These results with other sets of studies (Lundbaek et al., 2005, Greisen et al., 2011, Ashrafuzzaman et al., 2007 Sun et al., 2020) altogether raise our general understanding of the universality of the effects of MAAs (independently or their binary combinations) on bilayer physical properties. We can thus state that despite any MAA (peptides, CDs or amphiphiles) may not disrupt lipid bilayer electrical barrier properties, especially at low nM-μM concentrations in membrane bathing aqueous buffer, it may still exert effects on other types of bilayer physical properties and thus subsequently regulate the effects of other MAAs on cell membrane. Here we have addressed this phenomenon considering the effects of two nonchannel forming amphiphiles that alter bilayer physical properties but the membrane conductance on specifically a toroidal type pore forming CD candidate molecules. This study will be considered important in understanding membrane based effects of chemotherapy drugs (specifically) and other general drugs, thus may provide feedback information to aid in designing novel drugs for various diseases where the drugs need to interact with or cross through cell membranes and reach at certain intracellular targets.

## Declaration of Competing Interest

None
