# Supplementary material for: Amphiphiles capsaicin and triton X-100 regulate the chemotherapy drug colchicine’s membrane adsorption and ion pore formation potency
Source: Saudi J Biol Sci. 2021 Feb 21;28(5):3100–9. doi: 10.1016/j.sjbs.2021.02.054 (PMC8117037; doi:10.1016/j.sjbs.2021.02.054)
Supplement: Supplementary data 1 [file mmc1.docx]

**Supp. Fig. 1** GS effects on CD pores. GS increases the CD-induced ion pore induction potency. TCC (90 μM) alone permeabilizes lipid bilayer membranes by inducing nonzero current events (top panel). Middle and bottom panel represent the CD induced current events being influenced by the effects of 100 nM and 200 nM GS, respectively, added into the aqueous phases in both chambers. Both traces were filtered at 20kHz. POPE:PS:PC=5:3:2, 500 mM NaCl+90 μM TCC (*cis* side of the membrane), 500 mM NaCl+0 μM TCC (*trans* side of the membrane), 100 mV, pH 7.4. 200 nM GS alone could not induce ion conductance across the membrane (Ashrafuzzaman et al., 2008).

**Supp. Fig. 2** TX100 and Cpsn (not shown here) produce similar increases in gA channel activity. 60 s current traces recorded in the absence and presence of either 10 µM TX100 or 30 μM Cpsn. The experiments were done using two different gA analogues, AgA(15) and gA^−^(13), which were added to both sides of the bilayer. AgA(15) and gA^−^(13) channels can be distinguished by their current transition amplitudes (indicated by the two horizontal lines in the top trace): AgA(15) -----; and gA^−^(13) ^. . . . .^. The calibration bars in the top trace apply to all 4 traces. DOPC, 1.0 M NaCl, pH 7.0, 200 mV. The data (yet to be published) were produced at Weill Medical College of Cornell University by Md. Ashrafuzzaman at the laboratory of Olaf Sparre Andersen.

**Supp. Fig. 3** Amphiphile-induced changes in alamethicin channel activity. Cpsn, TX100 and rTX100 increase Alm channel activity. Top four records: 40 s recorded before the addition of amphiphile and after the addition of the indicated amphiphile. The calibration bars in the top trace apply to all four traces. Bottom four traces show the effect of the amphiphiles at higher resolution; calibration bars in the control trace segment apply to all the trace segments. The stippled lines denote different current levels; they do not vary with amphiphile addition (see also Table 1). DOPC, 1.0 M NaCl, pH 7.0, 150 mV. The data (yet to be published) were produced at Weill Medical College of Cornell University by Md. Ashrafuzzaman at the laboratory of Olaf Sparre Andersen.

**Supp. Fig. 4** pH effects on CD-induced pore formation. A may be considered as the pore activity.
